# Effect of Dietary *Astragalus polysaccharides* (APS) on the Growth Performance, Antioxidant Responses, Immunological Parameters, and Intestinal Microbiota of Coral Trout (*Plectropomus leopardus*)

**DOI:** 10.3390/microorganisms12101980

**Published:** 2024-09-30

**Authors:** Xiaoqi Hao, Heizhao Lin, Ziyang Lin, Keng Yang, Jing Hu, Zhenhua Ma, Wei Yu

**Affiliations:** 1 Key Laboratory of South China Sea Fishery Resources Exploitation & Utilization, Ministry of Agriculture and Rural Affairs, South China Sea Fisheries Research Institute, Chinese Academy of Fishery Sciences, Guangzhou 510300, China; haoxiaoqi@scsfri.ac.cn (X.H.); linheizhao@scsfri.ac.cn (H.L.); linziyang@scsfri.ac.cn (Z.L.); yangkeng66@163.com (K.Y.); hujing@scsfri.ac.cn (J.H.); zhenhua.ma@scsfri.ac.cn (Z.M.); 2 Ocean College, Hebei Agricultural University, Qinhuangdao 066003, China; 3 Shenzhen Base of South China Sea Fisheries Research Institute, Chinese Academy of Fishery Sciences, Shenzhen 518121, China; 4 Key Laboratory of Efficient Utilization and Processing of Marine Fishery Resources of Hainan Province, Sanya Tropical Fisheries Research Institute, Sanya 572426, China

**Keywords:** herbal medicine, dietary supplement, antioxidant status, immunomodulation, intestinal health, marine fish

## Abstract

The potential effects of *Astragalus polysaccharides* (APS) were evaluated in coral trout (*Plectropomus leopardus*). Five APS levels (0%, 0.05%, 0.10%, 0.15%, and 0.20%) were added to the diet of coral trout, and a 56-day growth trial (initial weight 18.62 ± 0.05 g) was conducted. Dietary APS enhanced growth performance, with the highest improvement observed in fish fed the 0.15% APS diet. This concentration also enhanced the antioxidant capacity and immunomodulation of the fish by regulating the expression of genes associated with antioxidant enzymes and immune responses. Intestinal microbiota analysis revealed that APS supplementation significantly increased the Chao1 index and relative abundance of beneficial bacteria (*Firmicutes* and *Bacillus*). A high level of APS (0.20%) did not provide additional benefits for growth and health compared to a moderate level (0.15%). These findings indicate that an optimal APS dose promotes growth, enhances antioxidant activity, supports immune function, and improves intestinal microbiota in coral trout. Based on a cubic regression analysis of the specific growth rate, the optimal APS level for the maximal growth of coral trout was determined to be 0.1455%.

## 1. Introduction

The coral trout, *Plectropomus leopardus,* is a marine creature with high economic and ornamental value in China and is prized for its delicious meat and rich nutritional content [1,2]. As high-density intensive farming becomes more common, cultivated coral trout have become more susceptible to diseases, in contrast to their wild counterparts [3,4]. Although antibiotics are frequently employed to treat these infections, their excessive use can result in environmental pollution and the development of antibiotic-resistant bacteria [5]. To address these concerns, aquaculture is gradually phasing out harmful traditional treatments [6]. Therefore, developing environmentally friendly and nutritionally balanced feed ingredients with immunomodulatory properties is crucial.

*Astragalus polysaccharides* (APS) are natural active compounds derived from *Astragalus*, a traditional herbal medicine that has been used as an immune enhancer in China for nearly 2000 years [7,8]. Over the past few decades, dietary supplementation with APS has been widely reported to improve the nutritional status and physiological conditions of mammals, livestock, and humans. [7,9,10,11,12,13]. Recent studies have also reported that APS can enhance the growth performance, antioxidant capacity, and immune function of aquatic species, such as turbot (*Scophthalmus maximus*) [14], largemouth bass (*Micropterus salmoides*) [15], white shrimp (*Litopenaeus vannamei*) [16], Furong crucian carp (*Furong carp*♀ × *red crucian carp*♂) [17], crucian carp (*Carassius auratus*) [18], Chinese mitten crab (*Eriocheir sinensis*) [19], and pearl gentian grouper (♀*Epinephelus fuscoguttatus* × ♂*Epinephelus lanceolatus*) [20]. Supplementation with APS in diets significantly increased growth (weight gain, specific growth rate (SGR), and feed conversion ratio) and immune (phagocytic activity, respiratory burst activity, plasma lysozyme (LZ), and bactericidal activity) parameters in Nile tilapia (*Oreochromis niloticus*) [21]. Additionally, dietary APS markedly increased the superoxide dismutase (SOD), catalase (CAT), and glutathione peroxidase (GSH-Px) activities in red claw crayfish (*Cherax quadricarinatus*) [22] and northern snakehead (*Channa argus*) [23], while reducing the malondialdehyde (MDA) levels. In addition to enhancing growth, antioxidant capacity, and immunity, APS also influences the digestive systems of aquatic animals. Studies have reported that dietary APS positively influences the activity of digestive enzymes and intestinal structure in grass carp (*Ctenopharyngodon idellus*) [24], large yellow croaker (*Larimichthys crocea*) [7], and Nile tilapia [21]. Furthermore, APS can enhance growth by improving intestinal health and modulating gut microbiota [25].

Previous investigations have primarily focused on the health benefits of APS for fish, and the potential negative outcomes of its overuse are underexplored [26]. Moreover, its effects on coral trout growth have not been reported. In this study, we assessed the effects of APS on growth, antioxidant status, immunological parameters, intestinal morphology, digestive enzyme activity and flora, liver antioxidant and immune-related enzyme activities, and gene expression in coral trout. We also determined the optimal APS dosage and explored the negative consequences of exceeding this amount. These results are expected to provide a theoretical basis for dietary APS supplementation in coral trout.

## 2. Materials and Methods

### 2.1. Materials and Experimental Diets

APS, with a purity of 91.5%, was obtained from Beijing Shoutianzhixin Technology Co., Ltd. (Beijing, China). Following the procedure outlined by Yu et al. [27], five diets were prepared with APS contents of 0%, 0.05%, 0.10%, 0.15%, and 0.20%; details are provided in Table S1. Detailed operational procedures are outlined in Supplementary Materials (Method S1).

### 2.2. Fish Management

The aquaculture setup was a recirculating aquaculture system (RAS), which included 15 tanks of 1.0 m³ each. Three hundred fish were randomly dispersed across the tanks after acclimatization, with three replicates used for each diet. For 8 weeks, each experimental diet was hand-administered twice a day (at 8:30 and 16:30) until the fish reached apparent satiation. Water conditions were maintained at a temperature of 28.00 ± 2.00 °C, pH 7.15 ± 0.15, dissolved oxygen >7.00 mg/L, and ammonia nitrogen <0.05 mg/L.

### 2.3. Sampling

Fish were fasted 24 h before sampling and anesthetized using eugenol (50 mg/L; Jian Huashuo Spice Oil Co., Ltd., Jian, China). The total quantity and weight of fish in each tank were recorded. The weight and length of three fish from each cage were measured to calculate the morphology indices, and six fish were randomly selected from each replicate for sampling. Subsequently, liver samples were taken from six fish and preserved at −80 °C for gene expression analysis. The remaining liver tissues were also stored at −80 °C for enzyme activity analysis. The intact intestines of the three fish were removed for gut microbiome analysis. Midgut samples from three additional fish were obtained and soaked in 4% paraformaldehyde solution (Biosharp, Guangzhou, China) for histological tests, while the remaining intestinal tissues were stored at −80 °C for enzyme activity analysis.

### 2.4. Calculations

The weight gain rate (WGR, %), specific growth rate (SGR, %/day), survival rate (SR, %), viscerosomatic index (VSI, %), hepatosomatic index (HSI, %), and condition factor (CF, g/cm^3^) were determined using the following equations:

WGR = 100 × (final weight (g) − initial weight (g))/(initial weight (g))(1)

SGR = 100 × (ln (final weight (g)) − ln (initial weight (g)))/(days)(2)

SR = 100 × (final fish number)/(initial fish number)(3)

VSI = 100 × (visceral weight (g))/(body weight (g))(4)

HSI = 100 × (hepatic weight (g))/(body weight (g))(5)

CF = 100 × (body weight (g))/(body length (cm))^3^ (6)

### 2.5. Proximate Composition

Diet composition was determined as previously described [28]. Moisture content was determined in the oven (105 °C) until constant weight. Crude protein and crude lipid contents were determined using the Kjeldahl nitrogen and Soxhlet extractions, respectively. The diets were burned in a muffle furnace (550 °C) for the determination of the ash contents.

### 2.6. Enzyme Activity Analysis

Enzyme activities related to digestion, antioxidant defenses, and immune response were measured using commercial assay kits (Jiancheng, Ltd., Nanjing, China) according to the manufacturer’s guidelines and formulas included with the kits. Detailed operational procedures are outlined in Method S2.

### 2.7. Real-Time Quantitative PCR Assay

Table S2 lists the specific primers of target genes and the reference gene (*GAPDH*). The qPCR reaction was conducted using the SYBR Green Pro Taq HS Premix kit (Accurate Biotechnology Co., Ltd., Changsha, China). Detailed operational procedures are outlined in Method S3. Gene expression levels were measured following Livak and Schmittgen’s approach [29].

### 2.8. Mid-Gut Histological Observation

Intestinal sections were stained with H&E and photographed. Morphological parameters were determined using CaseViewer 2.4 software, as described by Xie et al. [30].

### 2.9. Gut Microflora

Based on the analysis of growth performance results, 0.15% APS was identified as the optimal level for coral trout in this study. Gut microbiota effects were analyzed by selecting samples from the control group and the groups with the highest SGR and WGR groups. Amplification of the hypervariable 16S rRNA gene V3-V4 regions was performed using the specific 341F (CCTAYGGGRBGCASCAG) and 806R (GGACTACNNGGGTATCTAAT) primers with unique barcodes for each sample. Purified amplification products were linked to sequencing junctions, and sequencing libraries were created. Sequencing was performed on an Illumina platform. Raw data underwent splicing, filtering, and chimera removal to generate the final effective dataset for evaluation of the characteristics of intestinal microbiota. DNA amplification and sequencing were conducted by Beijing Novogene Co., Ltd. (Beijing, China).

### 2.10. Statistical Analysis

Data were presented as mean ± standard error (mean ± SE). Statistical significance was set at *p* < 0.05. Statistical analysis was performed using SPSS 27.0 software employing a one-way ANOVA to compare datasets among groups. Duncan’s and LSD multiple comparison tests were utilized to evaluate significant differences between and within groups. The cubic regression model was applied to predict the optimal APS level for the SGR of coral trout, which was identified as the most effective model because of its low residual sum of squares and high goodness-of-fit (R^2^) values.

## 3. Results

### 3.1. Growth Performance

**Figure 1:**
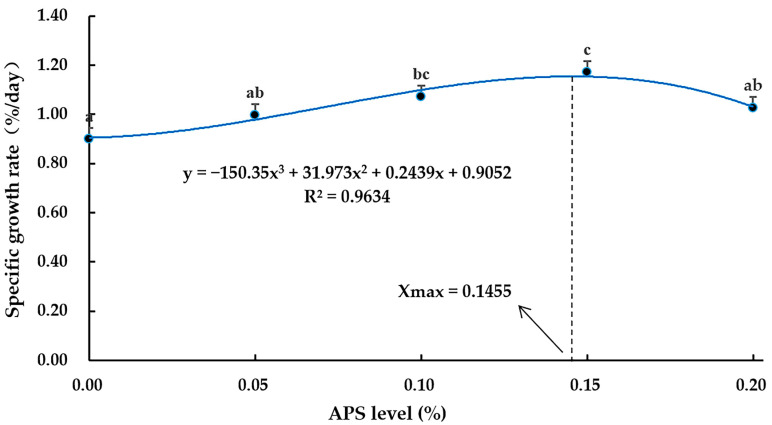
Prediction of the *Astragalus polysaccharides* (APS) dose required for maximal SGR of coral trout (*Plectropomus leopardus*) using cubic regression analysis. Values are shown as the mean ± SE (*n* = 3). Different letters indicate significant differences between the treatments.

As APS levels increased from 0.10% to 0.15%, the final body weight (FBW), WGR, and SGR in coral trout improved compared to the control (*p* < 0.05) (Table 1). However, the 0.20% APS group exhibited significantly lower FBW, WGR, and SGR than the 0.15% APS group (*p* < 0.05). SR, VSI, HSI, and CF were not significantly affected by different levels of APS supplementation (*p* > 0.05). The optimal level of dietary APS in the coral trout diet, based on SGR, was determined to be 0.1455% (Figure 1).

### 3.2. Digestive Enzyme Activities

Diets containing 0.10% to 0.15% APS significantly increased α-amylase and lipase activities compared to other diets (*p* < 0.05) (Table 2). No effect on chymotrypsin activity was observed owing to the dietary APS modifications (*p* > 0.05).

### 3.3. Gut Morphology

**Figure 2:**
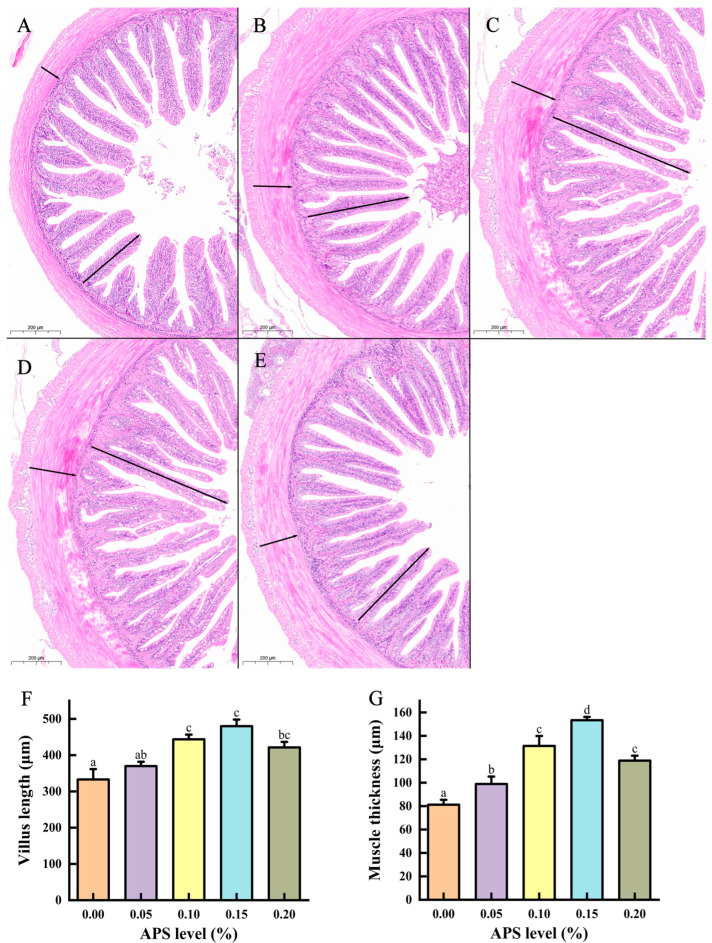
Intestinal morphology in coral trout (*Plectropomus leopardus*) fed different *Astragalus polysaccharides* (APS) dietary levels (long arrow, villus length; short arrow, muscle thickness). (**A**) 0% APS; (**B**) 0.05% APS; (**C**) 0.10% APS; (**D**) 0.15% APS; (**E**) 0.20% APS; (**F**) villus length; (**G**) muscle thickness. Scale bar, 200 μm. Values are shown as the mean ± SE (*n* = 3). Different letters indicate a significant difference.

Midgut villus length and muscle thickness were significantly increased in groups receiving 0.10%–0.20% APS (*p* < 0.05) compared to the control. Additionally, H&E-stained intestinal sections revealed no significant morphological alterations in the intestinal tissues of the test fish that received diets containing varying APS concentrations (Figure 2).

### 3.4. Liver Antioxidant Capacity

Superoxide dismutase (SOD) activity in the 0.10% to 0.15% APS groups and catalase (CAT) activity in the 0.15% APS group were significantly higher than those in the control group (*p* < 0.05) (Table 3). GSH-Px activity and total antioxidant capacity (T-AOC) peaked in the 0.15% APS group, showing significantly higher levels in the control and 0.20% APS groups (*p* < 0.05). Additionally, the MDA levels decreased significantly as the dietary APS dose increased (*p* < 0.05).

### 3.5. Liver Immune Function

Table 4 shows that alkaline phosphatase (AKP) activity, complement 4 (C4), and immunoglobulin M (IgM) levels were significantly higher in the 0.15% APS group compared to the control group (*p* < 0.05). The 0.10% to 0.20% APS groups exhibited higher acid phosphatase (ACP) and LZ activities compared to the control group (*p* < 0.05). The complement 3 (C3) levels were significantly increased in the 0.10% to 0.15% APS groups relative to both the control and 0.20% APS groups (*p* < 0.05).

### 3.6. Relative mRNA Expression in the Liver

**Figure 3:**
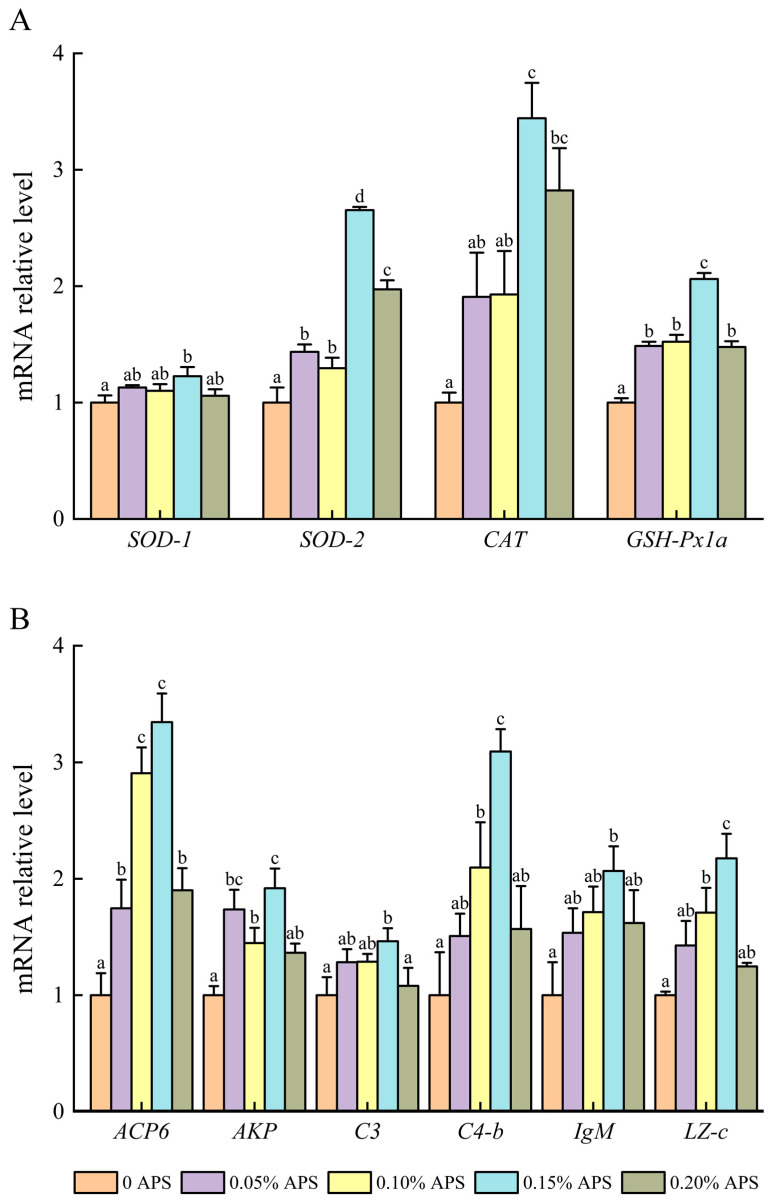
Expression of liver antioxidant enzyme (**A**) and immune indicator (**B**) genes in coral trout (*Plectropomus leopardus*) fed different *Astragalus polysaccharides* (APS) dietary levels. Values are shown as the mean ± SE (*n* = 3). The full names of the PCR target genes (*SOD-1*, *SOD-2*, *CAT*, *GSH-Px1a*, *ACP6*, *AKP*, *C3*, *C4-b*, *IgM*, *LZ-c*, and *GAPDH*) are provided in Table S2. Different letters indicate a significant difference.

Figure 3 shows the antioxidant and immune-related gene expression in coral trout across the different APS groups. The highest relative expression levels of *SOD-2*, *GSH-Px1a*, and *ACP6* were observed in the APS-treated groups, with significant differences from the control (*p* < 0.05). The 0.15% APS group exhibited significantly higher expression levels of these genes, along with *AKP*, *C3*, *C4-b*, and *LZ-c*, compared to the control and 0.20% APS groups (*p <* 0.05). Moreover, the expression levels of *SOD-2*, *GSH-Px1a*, *ACP6*, *AKP*, *C4-b*, and *LZ-c* were significantly increased in the 0.10% and 0.15% APS groups compared to the control (*p* < 0.05).

### 3.7. Gut Microbiota

#### 3.7.1. Intestine Microbial Diversity

**Figure 4:**
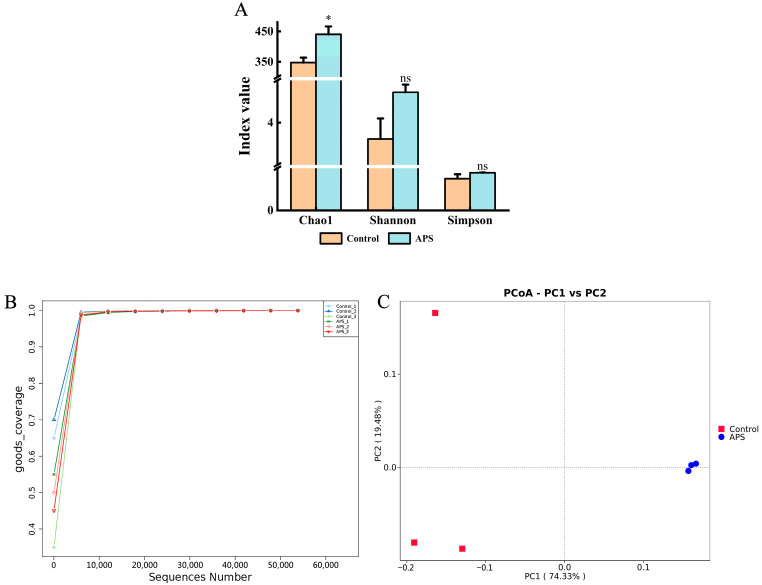
Alpha-diversity and beta-diversity of the gut bacterial community in coral trout (*Plectropomus leopardus*) fed different *Astragalus polysaccharides* (APS) dietary levels. (**A**) Chao1, Shannon, and Simpson index. Values are shown as the mean ± SE (*n* = 3) via the student’s two-tailed *t*-test. Different symbols (*, *p* < 0.05; ns, not significant) indicate significant differences (*p* < 0.05). (**B**) Rarefaction curve of the intestines in coral trout fed different APS dietary levels. (**C**) PCoA (Principal Co-ordinates Analysis) of the intestines in coral trout fed different APS dietary levels based on weighted UniFrac distances. Control, 0% APS group; APS, 0.15% APS group.

Figure 4A shows the alpha-diversity indices, including Chao 1, Shannon, and Simpson. The Chao1 index of the 0.15% APS group was considerably higher compared to the control group (*p* < 0.05). No significant differences in the Shannon and Simpson indices were observed (*p* > 0.05). The rarefaction curves of species in the intestines of the two groups indicated that the sequencing depth was sufficient (Figure 4B). A principal co-ordinates analysis (PCoA) plot based on a weighted UniFrac distance matrix was executed to show a clear separation between intestinal microbial communities in coral trout fed with APS and control diets, indicating significant compositional differences (Figure 4C).

#### 3.7.2. Intestine Microbial Composition

**Figure 5:**
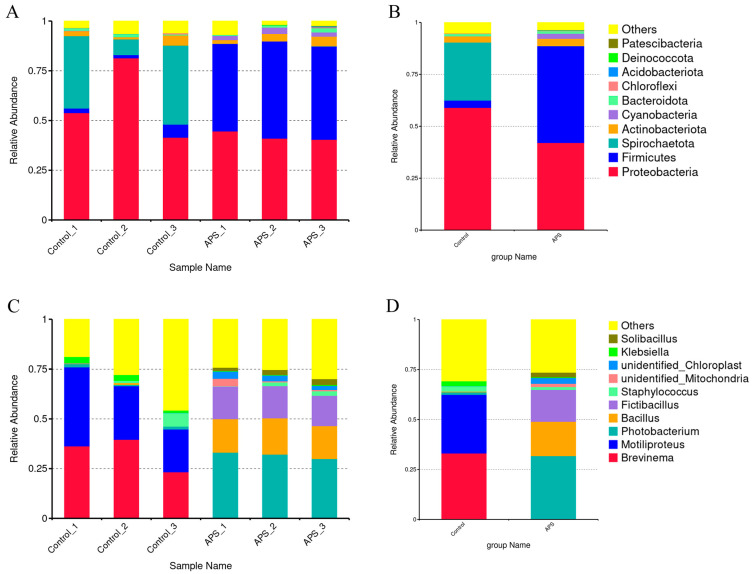
Bacterial compositions in coral trout (*Plectropomus leopardus*) fed different *Astragalus polysaccharides* (APS) dietary levels. (**A**,**B**) Distribution of the top 10 microbial phylum levels in different groups, with the legend shown on the right. (**C**,**D**) Distribution of the top 10 microbial genus levels in different groups, with the legend shown on the right. Control, 0% APS group; APS, 0.15% APS group.

Figure 5 presents the relative abundance of intestinal flora, providing an overview of the microbial composition at both the phylum and genus levels. According to the taxonomic results at the phylum level (Figure 5A,B, top 10), in the control group, *Proteobacteria* was the most abundant, followed by *Spirochaetota*, *Firmicutes*, and *Actinobacteriota*, at 58.95%, 27.96%, 3.50%, and 2.92%, respectively. Conversely, in the 0.15% APS group, *Firmicutes* was the predominant phylum, followed by *Proteobacteria*, *Actinobacteriota*, and *Cyanobacteria* (46.58%, 42.04%, 3.51%, and 2.46%, respectively). Based on the taxonomic results at the genus level (Figure 5C,D, top 10), *Brevinema* and *Motiliproteus* (33.11% and 29.35%, respectively) were more dominant in the control group, whereas *Photobacterium*, *Bacillus*, and *Fictibacillus* (31.67%, 17.18%, and 15.95%, respectively) were more abundant in the 0.15% APS group.

## 4. Discussion

The current study demonstrates that dietary supplementation with 0.15% APS resulted in the highest WGR and SGR in coral trout, suggesting that APS positively influences coral trout growth. These findings align with previous research on Nile tilapia [21] and large yellow croaker [7]. The growth-promoting effects of APS on coral trout can be attributed to two key factors. First, the appropriate APS dosage stimulated increased α-amylase and lipase activities in coral trout. Digestive enzyme activity is often considered an indirect indicator of fish growth [31]. In the present study, we identified a positive correlation between digestive enzyme activity and growth markers (WGR and SGR), potentially explaining the growth-promoting effects of APS on coral trout. Similarly, studies on largemouth bass (*Micropterus salmoides*) [32], large yellow croaker [7], crucian carp [18], giant freshwater prawn (*Macrobrachium rosenbergii*) [33], and spotted sea bass (*Lateolabrax maculatus*) [34] demonstrated that dietary APS supplementation significantly enhanced digestive enzyme activities. Additionally, intestinal morphological characteristics are essential for evaluating the digestive capacity and intestinal health of fish [35]. Supplementation with 0.1% to 0.15% APS has been shown to increase intestinal villus length and muscle thickness, which could also explain the accelerated growth of coral trout. This result is consistent with the findings of Liu et al. [7] and Duan et al. [32], who observed similar effects in large yellow croaker and largemouth bass, respectively. However, a high dose of APS (0.20%) did not further improve WGR and SGR compared to a moderate dose (0.15%), indicating potential immunosuppressive effects at higher doses, which may suppress growth performance [18,26]. In this study, the optimal APS dosage (0.1455%) was established according to the SGR of coral trout. Studies with similar results have indicated that the growth of aquatic animals initially increases with enhanced APS supplementation but eventually plateaus and declines [7,17,19]. Despite these findings, the role of APS in enhancing the growth of aquatic animals is not fully understood. Previous studies have shown that feeding APS to sea cucumbers (*Apostichopus japonicus*) [9] and red claw crayfish [22] did not enhance their growth. These studies demonstrate that the growth impact of APS in aquatic animals was contingent on the species, dosage, and experimental conditions. Thus, cultivation strategies that are carefully adapted to the needs of each fish species should be developed to enhance growth performance.

APS is also considered a potent antioxidant, potentially effective in boosting antioxidant enzyme activity and reducing oxidative stress in aquatic species [26,36]. MDA is a major and most extensively studied biomarker of lipid peroxidation [7,37]. In this study, the antioxidant capacity of coral trout was notably improved with APS supplementation, as evidenced by increased activities of SOD, CAT, GSH-Px, and T-AOC, along with a decrease in MDA levels, particularly in the 0.15% APS group. Earlier research has demonstrated similar outcomes, demonstrating that APS supplementation could boost SOD, CAT, and T-AOC activities in large yellow croaker [7] and largemouth bass [32], and significantly reducing MDA content. Yu et al. [26] also observed that adding APS to the diet of Asian seabass (*Lates calcarifer*) significantly enhanced SOD, CAT, and GSH-Px activities, increased T-AOC levels, and reduced MDA contents, though the maximum dose of 0.20% APS did not yield the highest total antioxidant capacity. In line with their findings, our study found that when the APS dose was increased to 0.2%, the GSH-Px activity and T-AOC contents in the liver were significantly lower than those observed in the 0.15% group, suggesting that higher levels of APS may not provide additional antioxidant benefits for coral trout.

ACP and AKP are crucial for enhancing fish resistance to microbial pathogens [38]. Previous research by Zhang et al. [39] on large yellow croaker (*Larimichthys crocea*) and by Song et al. [9] on sea cucumber demonstrated that APS could enhance the activities of non-specific immune enzymes, such as ACP and AKP. C3, C4, IgM, and LZ serve as key indicators of fish immunity [40]. APS significantly enhanced the aforementioned immunological parameters in *C. argus* [23] and *L. calcarifer* [26]. Consistent with these findings, the present study found that APS significantly boosted ACP, AKP, and LZ activity along with C3, C4, and IgM levels in coral trout, showing the highest levels in the 0.15% APS group. However, when the APS dose was increased to 0.2%, the C3, C4, and IgM levels significantly dropped compared with those observed in the 0.15% group. Similarly, Pu et al. [16] and Wu [18] found that high levels of *Astragalus membranaceus* polysaccharide (AMP) did not further enhance the innate immune response in white shrimp and crucian carp compared to moderate AMP levels. These results indicated that coral trout do not require higher APS levels.

To better understand the effects of APS supplementation on coral trout, we analyzed mRNA levels in the liver and found that diets containing 0.15% APS significantly upregulated the expression of *SOD-1*, *SOD-2*, *CAT*, *GSH-Px1a*, *ACP6*, *AKP*, *LZ-c*, *IgM*, *C3*, and *C4-b*. While increased mRNA levels partially elevated enzyme activity, the changes in enzyme activity did not always align with mRNA expression [41]. Our study on coral trout revealed that only the liver levels of CAT, GSH-Px, ACP, LZ, C3, and C4 were consistent with the trends in mRNA expression. In groups fed 0.2% APS, the expression levels of *SOD-2*, *GSH-Px1a*, *ACP6*, *AKP*, *C3*, *C4-b*, and *LZ-c* were notably lower than those in groups fed with 0.15% APS. This alteration corresponded to the trend observed in enzyme activity changes in the liver, implying that an excess of APS reduced its effectiveness.

Intestinal microbes are crucial for nutrient digestion, absorption, and the overall health of fish [42]. Research has shown that herbal medicines can stimulate appetite and influence microbial communities within animals, with greater diversity in the intestinal microbiota leading to higher functional stability [8,43,44]. We found that the APS-fed groups had higher diversity indices (Chao1, Shannon, and Simpson) than the control group, suggesting that APS supplementation supports intestinal homeostasis. Additionally, PCoA analysis with weighted UniFrac distances and differences in intestinal bacterial composition in the control and APS groups indicated that dietary APS modified the intestinal microbiota in coral trout. Therefore, a certain amount of APS enhances intestinal health.

APS can benefit gut microbial communities by enhancing the growth of beneficial bacteria while reducing the risk of bacterial and viral infections [19,24]. At the phylum level, most species within *Proteobacteria* and *Spirochaetota* act as conditional pathogens [44,45]. The study results indicated that the relative abundances of *Proteobacteria* and *Spirochaetota* in the APS group were lower than those in the control group, suggesting that dietary APS supplementation reduces the risk of intestinal diseases in fish. Conversely, the abundance of *Firmicutes*, which play a critical role in the hydrolysis of maltose and trehalose, increased under the APS diet [46]. *Firmicutes* species are linked to butyrate production, which is crucial for gut microbiota-immune system communication, prevents intestinal infections by inhibiting pathogen adhesion to aquatic surfaces, and stimulates the innate immune response [47,48,49]. The observed increase in *Firmicutes* and the decrease in *Proteobacteria* and *Spirochaetota* abundance suggest that APS supplementation can potentially enhance intestinal health by mitigating the risk of intestinal diseases in fish. The *Bacillus* genus is widely used as a probiotic in aquaculture due to its ability to promote gut health and inhibit pathogenic bacteria [43,50,51]. *Brevinema*, a potential pathogen in the *Spirochaetota* phylum, was found to decrease in abundance in pompano (*Trachinotus ovatus*) as glycerol monolaurate levels increased [52], consistent with our findings. At the genus level, APS supplementation boosted beneficial bacteria such as *Bacillus* in the intestines of coral trout while suppressing harmful bacteria such as *Brevinema*, thereby optimizing the gut microbial composition and structure. These results suggest that APS supplementation may have a favorable effect on the intestinal health of coral trout. However, there is a limitation in the present study in that the gut microbiome of slow-growing fish has not been analyzed. Studying both high-growth and slow-growing fish could reveal how APS impacts the gut microbiome across different growth rates. Future research should investigate how APS may influence the gut microbiome at varying growth rates.

## 5. Conclusions

This study demonstrates that adding 0.15% APS can improve the growth, antioxidant capacity, immune function, and intestinal health of coral trout. However, compared with a moderate level of APS (0.15%), a high level of APS (0.20%) did not improve the efficiency of APS on the growth and health of coral trout. Therefore, 0.15% APS was optimal for coral trout growth. Furthermore, cubic regression analysis determined that the optimal APS level for maximum coral trout growth was 0.1455%. The study findings offer fresh perspectives on the underlying mechanisms of APS use in coral trout feed.

## Figures and Tables

**Table 1:** Growth performance in coral trout (*Plectropomus leopardus*) fed different *Astragalus polysaccharides* (APS) dietary levels.

Parameters	Diets (APS %)				
	0	0.05	0.10	0.15	0.20
IBW (g)	18.61 ± 0.11	18.49 ± 0.17	18.71 ± 0.07	18.50 ± 0.07	18.79 ± 0.13
FBW (g)	30.82 ±0.41 ^a^	32.34 ±0.86 ^ab^	34.12 ± 0.70 ^bc^	35.67 ± 0.73 ^c^	33.38 ± 0.57 ^b^
WGR (%)	65.60 ± 1.50 ^a^	74.95 ± 6.04 ^ab^	82.34 ± 4.41 ^bc^	92.86 ± 4.52 ^c^	77.67 ± 3.85 ^ab^
SGR (%)	0.90 ± 0.02 ^a^	1.00 ± 0.06 ^ab^	1.07 ± 0.04 ^bc^	1.17 ± 0.04 ^c^	1.03 ± 0.04 ^ab^
SR (%)	96.67 ± 1.67	95.00 ± 2.89	100.00 ± 0.00	100.00 ± 0.00	96.67 ± 1.67
VSI (%)	4.87 ± 0.13	4.99 ± 0.11	5.17 ± 0.10	5.21 ± 0.12	5.19 ± 0.18
HSI (%)	1.04 ± 0.04	1.05 ± 0.09	1.08 ± 0.06	1.09 ± 0.04	1.06 ± 0.04
CF (%)	2.17 ± 0.04	2.19 ± 0.06	2.22 ± 0.03	2.24 ± 0.06	2.12 ± 0.03

Values are shown as the mean ± SE (*n* = 3). IBW, initial body weight; FBW, final body weight; WGR, weight gain rate; SGR, specific growth rate; SR, survival rate; VSI, visceral somatic index; HSI, hepatic somatic indices; CF, condition factor. Different letters indicate significant differences between the treatments.

**Table 2:** Digestive enzyme activities in coral trout (*Plectropomus leopardus*) fed different *Astragalus polysaccharides* (APS) dietary levels.

Parameters	Diets (APS %)				
	0	0.05	0.10	0.15	0.20
α-Amylase (U/mg prot)	0.62 ± 0.05 ^a^	0.70 ± 0.04 ^ab^	0.78 ± 0.04 ^b^	0.85 ± 0.06 ^b^	0.75 ± 0.04 ^ab^
Lipase (U/g prot)	1.34 ± 0.05 ^a^	1.47 ± 0.06 ^ab^	1.57 ± 0.09 ^b^	1.66 ± 0.07 ^b^	1.54 ± 0.04 ^ab^
Chymotrypsin (U/mg prot)	0.92 ± 0.06	0.98 ± 0.07	1.13 ± 0.05	1.16 ± 0.11	1.12 ± 0.09

Values are shown as the mean ± SE (*n* = 3). Different letters indicate significant differences between the treatments.

**Table 3:** Liver antioxidant ability in coral trout (*Plectropomus leopardus*) fed different *Astragalus polysaccharides* (APS) dietary levels.

Parameters	Diets (APS %)				
	0	0.05	0.10	0.15	0.20
SOD activity (U/mg prot)	41.66 ± 7.72 ^a^	56.84 ± 2.51 ^ab^	64.62 ± 6.81 ^b^	68.79 ± 3.17 ^b^	60.22 ± 8.51 ^ab^
CAT activity (U/mg prot)	7.73 ± 0.45 ^a^	8.12 ± 0.23 ^ab^	8.44 ± 0.57 ^ab^	9.26 ± 0.49 ^b^	8.07 ± 0.39 ^ab^
GSH-Px activity (U/mg prot)	26.80 ± 1.30 ^a^	29.63 ± 1.74 ^ab^	31.98 ± 1.18 ^b^	36.63 ± 1.47 ^c^	28.88 ± 1.03 ^ab^
T-AOC levels (U/mg prot)	0.74 ± 0.02 ^a^	0.78 ± 0.03 ^ab^	0.85 ± 0.02 ^bc^	0.91 ± 0.02 ^c^	0.80 ± 0.03 ^ab^
MDA content (nmol/mg prot)	6.44 ± 0.18 ^b^	5.89 ± 0.17 ^b^	4.94 ± 0.21 ^a^	4.69 ± 0.22 ^a^	4.84 ± 0.20 ^a^

Values are shown as the mean ± SE (*n* = 3). SOD, superoxide dismutase; CAT, catalase; GSH-Px, glutathione peroxidase; T-AOC, total antioxidant capacity; MDA, malondialdehyde. Different letters indicate significant differences between the treatments.

**Table 4:** Liver immune ability in coral trout (*Plectropomus leopardus*) fed different *Astragalus polysaccharides* (APS) dietary levels.

Parameters	Diets (APS %)				
	0	0.05	0.10	0.15	0.20
ACP activity (U/g prot)	441.91 ± 6.67 ^a^	509.27 ± 10.40 ^b^	535.59 ± 20.48 ^bc^	563.56 ± 17.67 ^c^	528.06 ± 9.25 ^bc^
AKP activity (U/g prot)	87.01 ± 2.96 ^a^	98.50 ± 4.57 ^ab^	99.70 ± 5.84 ^ab^	105.81 ± 6.99 ^b^	98.03 ± 3.15 ^ab^
C3 content (μg/mg prot)	142.54 ± 9.26 ^a^	150.56 ± 6.42 ^a^	188.37 ± 6.75 ^b^	191.58 ± 9.57 ^b^	157.58 ± 9.06 ^a^
C4 content (μg/mg prot)	27.21 ± 3.96 ^a^	34.16 ± 1.24 ^ab^	36.35 ± 2.80 ^ab^	39.18 ± 2.76 ^b^	27.70 ± 3.39 ^a^
IgM content (μg/mg prot)	49.03 ± 1.12 ^ab^	45.02 ± 1.67 ^a^	55.73 ± 3.42 ^bc^	60.40 ± 2.33 ^c^	45.43 ± 3.34 ^a^
LZ activity (μg/mg prot)	143.92 ± 5.47 ^a^	148.98 ± 3.70 ^ab^	164.79 ± 2.43 ^c^	165.55 ± 4.69 ^c^	161.48 ± 2.86 ^bc^

Values are shown as the mean ± SE (*n* = 3). ACP, acid phosphatase; AKP, alkaline phosphatase; C3, complement 3; C4, complement 4; IgM, immunoglobulin M; LZ, lysozyme. Different letters indicate significant differences between the treatments.

## Data Availability

The original contributions presented in the study are included in the article/Supplementary Materials. Further inquiries can be directed to the corresponding author.

## Supplementary Materials

The following supporting information can be downloaded at: https://www.mdpi.com/article/10.3390/microorganisms12101980/s1, Table S1: Composition and proximate chemical analysis of the experimental diets (expressed as % dry matter); Table S2: Primers and amplification conditions used for quantitative real-time PCR in this study; Method S1: Diet preparation; Method S2: Enzyme activity analysis; Method S3: Gene expression analysis.
