# Supplementary material for: Effect of Dietary Astragalus polysaccharides (APS) on the Growth Performance, Antioxidant Responses, Immunological Parameters, and Intestinal Microbiota of Coral Trout (Plectropomus leopardus)
Source: Microorganisms. 2024 Sep 30;12(10):1980. doi: 10.3390/microorganisms12101980 (PMC11509791; doi:10.3390/microorganisms12101980)
Supplement: Supplementary file 1 [file microorganisms-12-01980-s001.zip › microorganisms-3219933-supplementary.pdf]

Table S1. Composition and proximate chemical analysis of the experimental diets (expressed as % dry matter).

Ingredients (%)	Diets (APS %)				
	**0**	**0.05**	**0.10**	**0.15**	**0.20**
Fish meal	49.00	49.00	49.00	49.00	49.00
Soy protein concentrate	14.10	14.10	14.10	14.10	14.10
Soybean meal	10.80	10.80	10.80	10.80	10.80
Beer yeast powder	5.00	5.00	5.00	5.00	5.00
Wheat meal	11.68	11.68	11.68	11.68	11.68
Fish oil	5.00	5.00	5.00	5.00	5.00
Soybean lecithin	1.50	1.50	1.50	1.50	1.50
Choline chloride	0.50	0.50	0.50	0.50	0.50
Ethoxyquin	0.10	0.10	0.10	0.10	0.10
Vitamin premix [1]	0.20	0.20	0.20	0.20	0.20
Mineral premix [2]	0.12	0.12	0.12	0.12	0.12
Monocalcium phosphate	0.50	0.50	0.50	0.50	0.50
Astragalus polysaccharides	0.00	0.05	0.10	0.15	0.20
Microcrystalline cellulose	1.00	0.95	0.90	0.85	0.80
Betaine	0.50	0.50	0.50	0.50	0.50
Total	100.00	100.00	100.00	100.00	100.00
Proximate composition (% dry matter)					
Moisture	10.14	10.18	10.18	10.29	10.37
Crude protein	50.41	50.45	50.28	50.57	50.52
Crude lipid	11.25	11.55	11.49	11.36	11.25
Ash	9.89	10.00	10.09	9.95	10.00

[1] Vitamin premix (contains per 1 kg): vitamin A (6,000,000 IU); vitamin D_3 (1,000,000 IU); vitamin E (50,000 IU); vitamin K_3 (1,000 mg); vitamin B_1 (5,000 mg); vitamin B_2 (8,000 mg); vitamin B_6 (5,000 mg); vitamin B_{12} (500 mg); D-biotin (500 mg); D-pantothenic acid (1,000 mg); folic acid (1,000 mg); nicotinamide (30,000 mg); vitamin C (100,000 mg); inositol (80,000 mg); Zn (1,000 mg); Se (5 mg).
[2] Mineral premix (contains per 1 kg): (Cu 5 g); Fe (80 g); Zn (70 g); Mn (30 g); I (0.8 g); Se (0.4 g).

Table S2. Primers and amplification conditions used for quantitative real-time PCR in this study.

Target gene	Primer sequences (5′-3′)	Annealing temperature (◦ C)	Accession number	Product (bp)	Reference
GAPDH	F: GTGGTGCCAGCCAGAACA<br>R: TGTGTATCCCAGAATGCCCT	57.18<br>55.40	XM_033641056.1	233	[41]
SOD-1	F: TTATTTTGAGCAGGAGGATGGT<br>R: TTGTTGTGGGGATTGAAGTGA	53.95<br>53.66	AY035854.2	153	[41]
SOD-2	F: TAGCAAAGGGAGATGTGACGG<br>R: TTGTCGTAGCCCAGCCAG	57.57<br>57.18	XM_033647953.1	244	[41]
CAT	F: TGCGGGCGAATGTTTCTACT<br>R: TGGCATAATCTGGGTTGGTGG	55.40<br>57.57	XM_033635388.1	110	[41]
GSH-Px1a	F: GCCGCTACTCTGCCAAGG<br>R: ACAAACAAGGGGTGGGCA	59.46<br>54.90	XM_033625833.1	190	[41]
ACP6	F: TCATCAGAGCCCAACAGGAAG<br>R: AAAACCCCCAGAGCCATCA	57.57<br>55.16	XM_033615145.1	83	[41]
AKP	F: TCGCCCCAATGTTGAGTG<br>R: GGGGATGTAGTTCTGCTCGTG	54.90<br>59.52	XM_033627784.1	251	[41]
LZ-c	F: TGGCTAACTGGGTTTGTCTG<br>R: TTTGGGGTGCGGTCATCATT	55.40<br>55.40	XM_033647320.1	139	[41]
IgM	F: CGCCCCCTAACGGAAGTAAA<br>R: GCTGAATCTGTCCTCCAATAATCC	57.45<br>57.86	HQ007252.2	159	[41]
C3	F: GGTAGCAGTGACAAGCCAAGAC<br>R: AAGGGTGGGCAGGCGTT	59.54<br>57.02	HQ259061.1	271	[41]
C4-b	F: CGGGTTGTGCCAGACGA<br>R: ATTCTTACCGCCTTCAGTGG	57.02<br>55.40	XM_033640590.1	381	[41]

GAPDH: glyceraldehyde-3-phosphate dehydrogenase; *SOD-1*: copper-zinc superoxide dismutase; *SOD-2*: manganese superoxide dismutase; *CAT*: catalase; *GSH-Px1a*: glutathione peroxidase 1a; *ACP6*: acid phosphatase 6; *AKP*: alkaline phosphatase; *LZ-c*: lysozyme c; *IgM*: immunoglobulin M; *C3*: complement 3; *C4-b*: complement 4b.
References [41] are cited in the main text.

Method S1. Diet preparation.

Fish meal, soy protein concentrate, and soybean meal were utilized as protein sources, while fish oil and soybean lecithin were used as the main lipid sources. Diet ingredients, including fish meal, were ground using a powder mill (SF-320, Suzhou Pharmaceutical Machinery Co., LTD., Jiangsu, China) and subsequently sieved through a 40-mesh screen. The ingredients were carefully weighed according to the diet formulation. After uniformly mixing the constant components, the trace components were gradually added and thoroughly mixed. The mixture was thoroughly combined with soy lecithin and fish oil for 10 minutes. Then, water was added, and the mixture was blended in a mixer (SZ250, Guangzhou Xuzhong Food Co., LTD., Guangdong, China) for an additional 5 minutes. The mixture was processed using an extruder (F-26, South China University of Technology, Guangdong, China) and subsequently a granulator (G-500, South China University of Technology, Guangdong, China) to create pellets with a diameter of 2.5 mm and 3.00 mm. Finally, the prepared diets were air-dried and stored in plastic bags at -20 °C until use.

Method S2. Enzyme activity analysis.

The thawed liver and intestinal tissue samples were rinsed with pre-cooled 0.9% physiological saline solution (Biosharp, Guangzhou, China) to remove the tissue fluid, followed by drying with filter paper. Subsequently, the tissues were precisely weighed and 0.9% physiological saline solution was added to a volume nine times greater than the tissue mass. Then, a 10% tissue homogenate of the samples was prepared using a tissue homogenizer (TissueLyser II, Qiagen, Berlin, Germany) at 4°C. After centrifugation at 3500 r/min for 10 min, a homogenate supernatant was obtained to determine the biochemical indicators. Enzyme activities were measured using a microplate reader instrument (Infinite M200 Pro, Switzerland TECAN, Zurich, Switzerland). The levels of superoxide dismutase (SOD), catalase (CAT), glutathione peroxidase (GSH-Px), total antioxidant capacity (T-AOC), malondialdehyde (MDA), acid phosphatase (ACP), alkaline phosphatase (AKP), complement components (C3 and C4), immunoglobulin M (IgM), lysozyme (LZ), α-amylase, lipase, chymotrypsin, and total protein (TP) were measured using commercial assay kits (Jiancheng, Ltd., Nanjing, China). The assays were performed and formulas were calculated according to the instructions provided with the test kits.

Method S3. Gene expression analysis.

Total RNA was isolated from liver tissue using an RNA extraction solution (Servicebio Technology Co., Ltd., Wuhan, China). RNA quality was assessed using 1.0% agarose gel electrophoresis, and the Nanodrop 2000 was used to determine the RNA concentration and purity. Subsequently, RNA was reverse transcribed into cDNA with a SweScript All-in-One RT SuperMix for qPCR (Servicebio Technology Co., Ltd., Wuhan, China), and any remaining DNA was eliminated with gDNA Remover (Servicebio Technology Co., Ltd., Wuhan, China). Reverse transcription was performed on a regular PCR system with the following program settings: 25°C for 5 min, 42°C for 30 min, and 85°C for 5 s. The cDNA was then stored at -80°C for subsequent experiments.

Table S2 provides specific details on the qPCR primers, and the forward and reverse specific primers were synthesized by Beijing Ruibo Xingke Biotechnology Co., Ltd. (Beijing, China). PAGE purification was performed to ensure high purity. *GAPDH* was used as the internal reference gene of coral trout.

The qPCR reaction was executed using a real-time fluorescent quantitative PCR detection system (Likang CG-02, China) with a SYBR Green Pro Taq HS Premix kit (Accurate Biotechnology Co., Ltd., Changsha, China). The quantitative real-time PCR amplification mixture (15 μL) included 1.0 μL cDNA, 0.6 μL 10 μmol/L forward primer, 0.6 μL 10 μmol/L reverse primer, 5.3 μL RNase-free water, and 7.5 μL SYBR Green Pro Taq HS Premix (2×). The qPCR amplification process consisted of initial denaturation at 95°C for 30 s, followed by 40 cycles of 95°C for 5 s and 60°C for 30 s. The relative expression levels of the target genes were calculated using the $2^{-\Delta\Delta Ct}$ method described by Livak and Schmittgen [29].

References [29] are cited in the main text.
